# Cloning and characterization of a novel functional organic anion transporting polypeptide 3A1 isoform highly expressed in the human brain and testis

**DOI:** 10.3389/fphar.2022.958023

**Published:** 2022-09-02

**Authors:** Éva Bakos, Orsolya Német, Nóra Kucsma, Natália Tőkési, Bruno Stieger, Elisabeth Rushing, Anna-Mária Tőkés, Péter Kele, Gábor E. Tusnády, Csilla Özvegy-Laczka

**Affiliations:** ^1^ Institute of Enzymology, RCNS, Budapest, Hungary; ^2^ University Hospital Zürich, University of Zurich, Zürich, Switzerland; ^3^ Department of Pathology, Forensic and Insurance Medicine, Semmelweis University, Budapest, Hungary; ^4^ Institute of Organic Chemistry, RCNS, Budapest, Hungary

**Keywords:** organic anion transporting polypeptide (OATP), isoform, choroid plexus, testis, steroid transport, human brain

## Abstract

Organic anion transporting polypeptide 3A1 (OATP3A1, encoded by the *SLCO3A1* gene) is a prostaglandin, oligopeptide, and steroid/thyroid hormone transporter with wide tissue distribution, expressed, e.g., in the human brain and testis. Although the physiological importance of OATP3A1 has not yet been clarified, based on its expression pattern, substrate recognition, and evolutionary conservation, OATP3A1 is a potential pharmacological target. Previously, two isoforms of OATP3A1, termed as V1 and V2, have been characterized. Here, we describe the cloning and functional characterization of a third isoform, OATP3A1_V3. The mRNA of isoform V3 is formed by alternative splicing and results in an OATP3A1 protein with an altered C-terminus compared to isoforms V1 and V2. Based on quantitative PCR, we demonstrate the widespread expression of SLCO3A1_V3 mRNA in human organs, with the highest expression in the brain and testis. By generation of an isoform V3-specific antibody and immunostaining, we show that the encoded protein is expressed in the human choroid plexus, neurons, and both germ and Sertoli cells of the testis. Moreover, we demonstrate that in contrast to isoform V1, OATP3A1_V3 localizes to the apical membrane of polarized MDCKII cells. Using HEK-293 cells engineered to overexpress OATP3A1_V3, we verify the protein’s functionality and identify dehydroepiandrosterone sulfate as a novel OATP3A1 substrate. Based on their distinct expression patterns but overlapping functions, OATP3A1 isoforms may contribute to transcellular (neuro)steroid transport in the central nervous system.

## Introduction

Organic anion transporting polypeptides (OATPs) are solute carrier-type membrane proteins expressed in various tissues of animal species, including human epithelial and endothelial cells (Hagenbuch and Stieger, 2013). OATPs mediate the sodium- and ATP-independent exchange of anions and amphipathic molecules across the plasma membrane (Stieger and Hagenbuch, 2014). Several multispecific members of the OATP family are dedicated drug transporters, influencing the pharmacokinetics of their substrates, e.g., statins, chemotherapeutics, antivirals, and antihypertensives (Shitara et al., 2013). Such drug transporter OATPs include OATP1A2, OATP1B1, OATP1B3, and OATP2B1. OATP1A2 is ubiquitously expressed in the human body, with the most abundant expression in the blood–brain barrier and neurons, where it influences the penetration of substrates through the blood–brain barrier (Gao et al., 2000, Gao et al., 2005, Gao et al., 2015). Similar to OATP1A2, OATP2B1 has broad tissue expression, with the highest expression in the intestine and liver (McFeely et al., 2019). The most extensively studied OATPs are OATP1B1 and OATP1B3, with (almost) unique expression in hepatocytes (Konig et al., 2000, Kullak-Ublick et al., 2001). OATP1B1 and OATP1B3 play an important role in hepatic drug clearance; hence, inhibition of their function by drug–drug or food–drug interactions or increased hepatic uptake by their function may lead to unwanted side effects or even toxicity.

In the human genome, 12 OATP encoding genes, termed as *SLCO* (solute carrier for organic anion transporting polypeptides), are found. However, as a result of alternative transcription initiation or alternative splicing, from the 12 genes, more than 12 proteins are formed (Nagai et al., 2012, Thakkar et al., 2013, Knauer et al., 2013, Huber et al., 2007). One of the *SLCO* genes with multiple isoforms is *SLCO3A1*, encoding OATP3A1.

OATP3A1 (previously termed OATP-D) is a ubiquitously expressed membrane protein (Tamai et al., 2000). *SLCO3A1* mRNA has been detected in numerous fetal and adult tissues (Tamai et al., 2000), while at the protein level, OATP3A1 expression has been shown in the central nervous system (choroid plexus and neurons (Huber et al., 2007)) as well as in the testis (Huber et al., 2007), breast (Kindla et al., 2011) and heart (Atilano-Roque and Joy, 2017). Physiological substrates of OATP3A1 encompass estrone-3-sulfate (E1S), prostaglandin (PG) E1 and E2, vasopressin, and thyroxine (Bakos et al., 2020, Huber et al., 2007, Tamai et al., 2000). In addition to these endogenous substances, OATP3A1 is also able to transport drugs. Simvastatin (Atilano-Roque and Joy, 2017), the endothelin A receptor antagonist BQ-123, and the opioid analgesic peptide deltorphin II (Huber et al., 2007) are documented OATP3A1 substrates. However, because of the lack of exhaustive studies, the list of substrates and inhibitors of OATP3A1 may be incomplete. Recently, we have identified various coumarin-based dyes as substrates of OATP3A1 (Bakos et al., 2020). These dyes, in addition to the previously described indicator, fluorescein (Atilano-Roque and Joy, 2017, Patik et al., 2015), can help in identifying further molecules interacting with OATP3A1. Although its physiological role has not yet been clarified, based on its expression pattern and substrate recognition, OATP3A1 may be important in steroid hormone and drug uptake into the brain. In addition, a protective role of OATP3A1 against bile salts has been suggested (Pan et al., 2018). In their study, Pan and colleagues demonstrated increased efflux of taurocholate and glycocholate from OATP3A1-expressing cells (Pan et al., 2018). Moreover, an increased expression of OATP3A1 was detected in various tumors (Banerjee et al., 2015, Hays et al., 2013, Kindla et al., 2011, Wlcek et al., 2011), although its role in chemotherapy response is not yet clarified.

Upon cloning of OATP3A1 from the human brain, Huber et al. (2007) identified two alternatively spliced variants of OATP3A1. Isoform 1 encodes a 710-amino acid long protein, OATP3A1_V1, identical with the GenBank accession no. AB031050. Isoform 2 encodes a shorter, 692-amino acid long protein, OATP3A1_V2 (GenBank accession no. BC000585), having a different C-terminus compared to OATP3A1_V1. Huber et al. (2007) found similar ubiquitous expression of OATP3A1_V1 and OATP3A1_V2, and both proteins were able to transport PGE1 and PGE2, thyroxine, and the cyclic oligopeptides BQ-123 and vasopressin. However, the localization of the two isoforms was different, with OATP3A1_V1 being a basolateral transporter and OATP3A1_V2 localized apically.

In the current study, we describe the cloning and characterization of a third isoform of OATP3A1, which we designate as OATP3A1_V3. We show that OATP3A1_V3 has similar activity but distinct membrane localization as the first described OATP3A1_V1 isoform.

## Materials and methods

### Materials

^3^H-estrone-3-sulfate (48 Ci/mmol) and ^3^H-dehydroepiandrosterone*-*sulfate (60 Ci/mmol) were obtained from Perkin Elmer (Waltham, MA, US). The fluorescent dye (E)-1-(3-azidopropyl)-4-(2-(7-(diethylamino)-2-oxo-2H-chromen-3-yl)vinyl)pyridine-1-ium-3-sulfonate (ACPS) was synthetized, as described previously (Nagy et al., 2010). Tissue total RNAs were obtained from Agilent Technologies (Kromat, Hungary). Estrone-3-sulfate, benzbromarone, and all other materials**,** if not stated otherwise, were purchased from Sigma-Aldrich (Sigma, Merck, Darmstadt, Germany).

## Methods

### Generation of constructs

To clone the complete open reading frame of OATP3A1 variants, total RNA from the brain was reverse-transcribed, and the resulting cDNA was amplified by HF PCR (Phusion^®^ High-Fidelity PCR Kit; NEB, Ipswitch, MA, US), following the manufacturer’s instructions with the forward primer 5′ TAA​AGG​ATC​CGC​GGC​CGC​GCC​ACC​ATG​CAG​GGG​AAG​AAG​CCG and the following reverse primers:

OATP3A1_V1: 5′ CAT​GTC​TCG​AGA​CTA​GTA​AGC​TTC​TAT​AAA​ACG​GAC​TCC​ATG, OATP3A1_V2: 5′ CAT​GTC​TCG​AGA​CTA​GTA​AGC​TTT​CAA​AGT​AGA​CTC​TGT​GG, OATP3A1_V3: 5′ CAT​GTC​TCG​AGA​CTA​GTA​AGC​TTT​CAG​CTT​CTC​ACA​AAG​GA.

After digestion with BamHI and SpeI enzymes (NEB, Ipswitch, MA, US), the PCR fragments were cloned to the corresponding sites of the pRRL-CMV-MCS-IRES-ΔCD4 vector (Patik et al., 2018). The base order of the cDNAs in all constructs was verified by sequencing. Comparison of these sequences with the previously reported OATP3A1_V1, OATP3A1_V2 and the predicted X1-OATP3A1 (XM_005254889 in the NCBI database), renamed to OATP3A1_V3 in this study, revealed that all isoforms contain the E294D polymorphism (rs1517618).

### Quantitative real-time PCR

For analysis of mRNA expression levels, 1 μg of total RNA from each human tissue was reverse transcribed by random oligomers using the Maxima First Strand cDNA Synthesis Kit (Thermo Fisher Scientific, Waltham, MA, US), according to the manufacturer’s instructions, and used as templates for quantitative real-time PCR (qPCR) assays. For qPCR reactions, 50 ng of cDNA was applied in a 20 μl reaction volume in triplicates. Reactions were carried out using the PowerUp^TM^ SYBR Green Master Mix (Applied Biosystems, Life Technologies, Carlsbad, CA, US) with the following forward primers 5′ GCT​CAA​ATC​CTT​CGC​CTT​CAT​C and isoform-specific reverse primers: 5′ GGT​CAG​AGT​AGA​GGC​AAA​GAA​C (OATP3A1_V1) or 5′ CTG​GCT​CTG​ATG​TGG​TGC​T (OATP3A1_V3). qPCR measurements were performed on a StepOnePlus^TM^ platform (Thermo Fisher Scientific) using a standard protocol. To verify that there was no non-specific PCR product, a melting curve analysis was performed following the PCR cycling. Amplifications were repeated three times. The results were analyzed by StepOne software version 2.1. Ribosomal protein P0 (RPLP0) was used as an endogenous reference gene (with the following primers: RPLP0 forward primer: 5′ CGC​GGG​AAG​GCT​GTG​GTG​CTG​A and RPLP0 reverse primer: 5′ GGG​CAA​TGG​CAC​CAG​CAC​GGG). The relative expression levels were calculated by the comparative Ct (2^−ΔCt^) method.

### Generation of cell lines

The original HEK-293 and MDCKII cell lines were obtained from ATCC. OATP3A1_V1, V2, and V3 overexpression in HEK-293 and MDCKII cells was achieved by recombinant lentiviruses. Retroviral transductions of HEK-293 and MDCKII cells were performed, as described earlier (Patik et al., 2018). In order to generate mock cells, HEK-293 and MDCKII cells were transduced with lentiviruses containing only the pRRL-EF1-ΔCD4 vector. All cell lines were cultured in DMEM (Gibco, Thermo Fisher Scientific (Waltham, MA, US)) supplemented with 10% fetal bovine serum, 2 mM L-glutamine, 100 U/ml penicillin, and 100 μg/ml streptomycin.

### Generation of the antibody

For producing a polyclonal antibody against OATP3A1_V3, the following peptide CVLEATCAAGPQSLL containing the protein’s C-terminus was coupled with the side chain of the N-terminal cysteine to KLH and provided by CASLO ApS (Kongens Lyngby, Denmark). The antiserum was raised, as previously detailed (Stieger et al., 1994).

### Immunoblot detection of OATP3A1 variants

Expression of OATP3A1 variants was confirmed by Western blot, as described in Bakos et al. (2020). Whole-cell lysates of HEK-293 (5 μg) were loaded onto 7.5% SDS/PAGE gels and transferred onto PVDF membranes. Immunoblotting with appropriate antibodies was performed, as described in Bakos et al. (2020). The antibody recognizing all OATP3A1 variants (pan anti-OATP3A1) was purchased from Sigma-Aldrich (SAB 1304633). OATP3A1_V3 was detected by using the isoform-specific antibody (see the aforementioned antibody). The anti-rabbit peroxidase-conjugated donkey IgG secondary antibody was obtained from Jackson ImmunoResearch (Cambridgeshire, UK), and it was used at a dilution of 20,000x. Proteins were visualized using the Luminor Enhancer Solution kit by Thermo Fisher Scientific (Waltham, MA, US).

### Immunofluorescence staining

5 × 10^5^ HEK-293-OATP3A1 and mock cells were seeded onto poly-L-lysine–coated 8-well *μ*-slides (Ibidi, Germany). The following day, the cells were fixed with 4% (w/v) formaldehyde in phosphate-buffered saline (PBS) for 15 min, washed with PBS, and then incubated in blocking solution (0.5% (w/v) BSA, 0.5% fish gelatin, 0.1% (w/v) Triton X-100, and 5% (v/v) goat serum in PBS) for 1 h at RT. For staining of OATP3A1 variants, the cells were incubated with either the pan anti-OATP3A1 (1:500) or anti-OATP3A1_V3 (1:500) antibody overnight at 4°C. After washing with PBS, the cells were incubated with the AlexaFluor-488-conjugated anti-rabbit IgG antibody (Invitrogen, Thermo Fisher Scientific, Waltham, MA, US, 1:250) for 1 h. After immunolabeling, the cells were counterstained with 1 μM DAPI for 10 min.

For localization studies, polarized MDCKII cells were seeded (5 × 10^4^ cells/insert) on 6.5-mm Transwell filters (0.4 mm pore diameter, Corning Costar). The cells were cultured to confluency for 10 days and then fixed with 4% (w/v) formaldehyde. To label the lateral plasma membrane, an anti E-cadherin antibody (Abcam11512, Abcam, Cambridge, MA (1:250)) was used. For staining of OATP3A1, the pan anti-OATP3A1 (1:500) or the OATP3A1_V3 (1:500) antibody was applied in the blocking solution. AlexaFluor-488-conjugated anti-rabbit IgG and AlexaFluor-647-conjugated anti-rat IgG (Invitrogen, Thermo Fisher Scientific, 1:250) were used as secondary antibodies. Nuclei were labeled with DAPI (1 μM).

Imaging was carried out using a Zeiss LSM-710 inverted confocal microscope with 40× (or 63x)/1.4 Plan Apochromat oil immersion objective (Carl Zeiss, Jena, Germany). All images were processed by Zen 2010 software (Carl Zeiss microscopy, Jena, Germany).

### Immunohistochemical staining

Paraffin-embedded, formalin-fixed tissue sections of testis were obtained from a 62-year-old man. Each section was examined by a pathologist and confirmed to be appropriate for immunohistological studies. Tissue sections were deparaffinized in xylol and rehydrated through graded ethanol; then, each sample was treated with 10 mM Tris/EDTA pH 9.0 for 20 min at 96°C. To inactivate the endogenous peroxidases, the sections were incubated in 3% (v/v) H_2_O_2_ for 10 min. After blocking, tissues were incubated with the anti-OATP3A1_V3 antibody in the blocking solution overnight at 4°C. The following day, after washing with PBS, sections were incubated with anti-rabbit HRP-polymer (Novolink^TM^ Polymer, Leica Biosystems, Newcastle upon Tyne, UK) for 30 min and washed with PBS. Sections were stained with diaminobenzidine as the chromogen (Novolink^TM^ DAB) for 5 min, then washed with water, and finally, nuclei were counterstained with hematoxylin.

Immunohistochemical staining of OATP3A1_V3 was performed on sections of the human brain using the anti-OATP3A1_V3 antibody on an automated staining system (Ventana Benchmark) using the OptiView Kit, P1 (4 min) with 30 min of incubation and 1:100 dilution.

### Determination of ACPS uptake by flow cytometry

Uptake of ACPS (E)-1-(3-azidopropyl)-4-(2-(7-(diethylamino)-2-oxo-2H-chromen-3-yl)vinyl)pyridine-1-ium-3-sulfonate) in HEK-293 cells overexpressing OATP3A1 variants was measured, as described previously (Bakos et al., 2020). Briefly, 5 × 10^5^ cells were collected, following trypsinization, and preincubated in the presence or absence of 20 µM benzbromarone for 5 min at 37°C. The reaction was started by the addition of the fluorescent ACPS. Transport experiments were carried out in the uptake buffer (25 mM MES, 125 mM NaCl, 4.8 mM KCl, 1.2 mM CaCl_2_, 1.2 mM KH_2_PO_4_, 12 mM MgSO_4_, and 5.6 mM glucose; pH 7.4) for 10 min at 37°C. Uptake was stopped by the addition of five volumes of ice-cold PBS. Cellular fluorescence was determined in at least 10,000 living cells using an Attune Acoustic Focusing Cytometer (Applied Biosystems, Life Technologies, Carlsbad, CA, US).

### Measurement of [^3^H]E1S and [^3^H]DHEAS uptake

Transport experiments were performed as described previously (Bakos et al., 2020). Briefly, HEK-293 cells overexpressing OATP3A1 variants were trypsinized, and 1 × 10^6^ cells in suspension were pre-incubated in the presence or absence of 20 µM benzbromarone for 5 min at 37°C. The reaction was initiated by the addition of the radiolabeled substrates, ^3^H-estrone-3-sulfate ([^3^H]E1S) or [^3^H]DHEAS in uptake buffer (pH 5.5). After incubation at 37°C for 10 min (E1S) or 5 min (DHEAS), transport was stopped by adding ten volumes of ice-cold PBS. After pelleting the cells by centrifugation, the cells were suspended in PBS, and the cell-associated radioactivity was measured with a Wallac Liquid Scintillator Counter.

### Data and statistical analysis

All data are presented as means ± SD from at least three independent experiments. Determination of the kinetic parameters of substrate uptake and the unpaired Student’s *t*-test used for statistical analyses were performed by GraphPad Prism software (GraphPad, La Jolla, CA, United States).

## Results

### Cloning of the novel OATP3A1_V3 isoform

**FIGURE 1:**
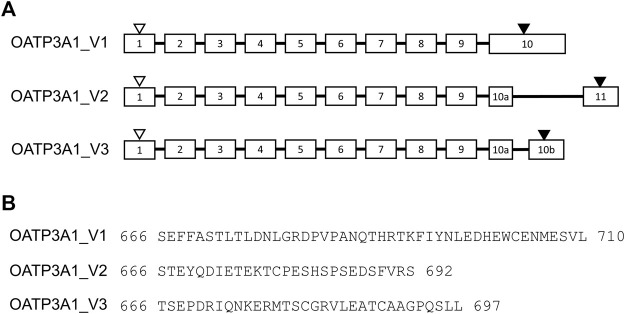
Exon–intron structure and amino acid sequence of the COOH terminus of OATP3A1 variants (OATP3A1_V1, OATP3A1_V2, and OATP3A1_V3). **(A)**. Exon–intron structure of OATP3A1 variants. Numbered boxes represent exons, and introns are shown as lines. The positions of the start and stop codon are indicated by open and filled triangles, respectively. The figure is drawn on the basis of the alignment of transcript mRNA of OATP3A1_V3 with the human genomic sequence. **(B)**. Alignment of the C-termini of OATP3A1 variants.

In addition to two previously identified and validated OATP3A1 isoforms, OATP3A1_V1 and OATP3A1-V2 (Huber et al., 2007), the NCBI protein database contains two additional potential OATP3A1 variants. Of these, X1-3A1-697 (XP_005254946.1), which we designate as OATP3A1_V3, may be a potential transporter with the predicted membrane topology containing 12 transmembrane helices. The sequence encoding this protein is computationally predicted and produced by NCBI’s genome annotation pipeline, but to date, there has been no experimental evidence for the existence of the protein or its transcript. Since the two previously characterized isoforms of OATP3A1 are highly expressed in the brain, we initially screened a brain-derived RNA library for expression of OATP_V3 mRNA. In these experiments, an mRNA containing the full-length open reading frame of OATP3A1_V3 was isolated. Sequencing revealed that OATP3A1_V3 has identical first nine exons with OATP3A1_V1. However, alternative splicing results in two alternative 10^th^ exons (10a and 10b) of OATP3A1_V3 mRNA and, consequently, a different C-terminus (aa 666–697) of the protein (Figure 1).

### Tissue expression pattern of OATP3A1_V3 mRNA

**FIGURE 2:**
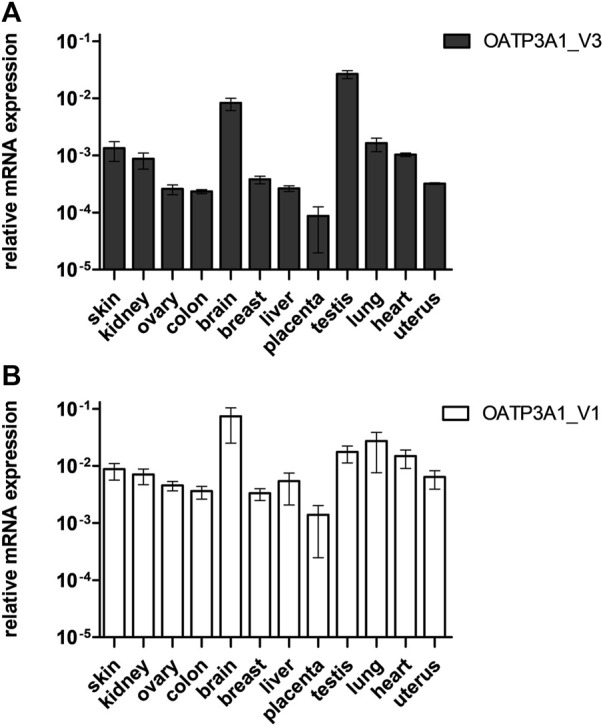
Relative mRNA expression of OATP3A1_V3 in human tissues. Relative expression levels of OATP3A1_V1 **(A)** and OATP3A1_V3 **(B)** were measured by quantitative real-time PCR and normalized to the mRNA expression of RPLP0 as endogenous control. cDNA was generated in three independent reactions, and all qPCRs were conducted in triplicates. Data are expressed as the mean ± SD values.

In order to analyze the tissue expression pattern of OATP3A1_V3, we performed quantitative RT-PCR on mRNA libraries obtained from 12 different organs. As shown in Figure 2A, the expression of OATP3A1_V3 mRNA could be confirmed in every organ, with the highest expression in the brain and testis. The observed, ubiquitous expression of OATP3A1_V3 is similar to that of OATP3A1_V1 (Figure 2B), although generally at a lower level in all tissues than OATP3A1_V1.

### Immunolocalization of OATP3A1_V3 using an isoform-specific antibody

**FIGURE 3:**
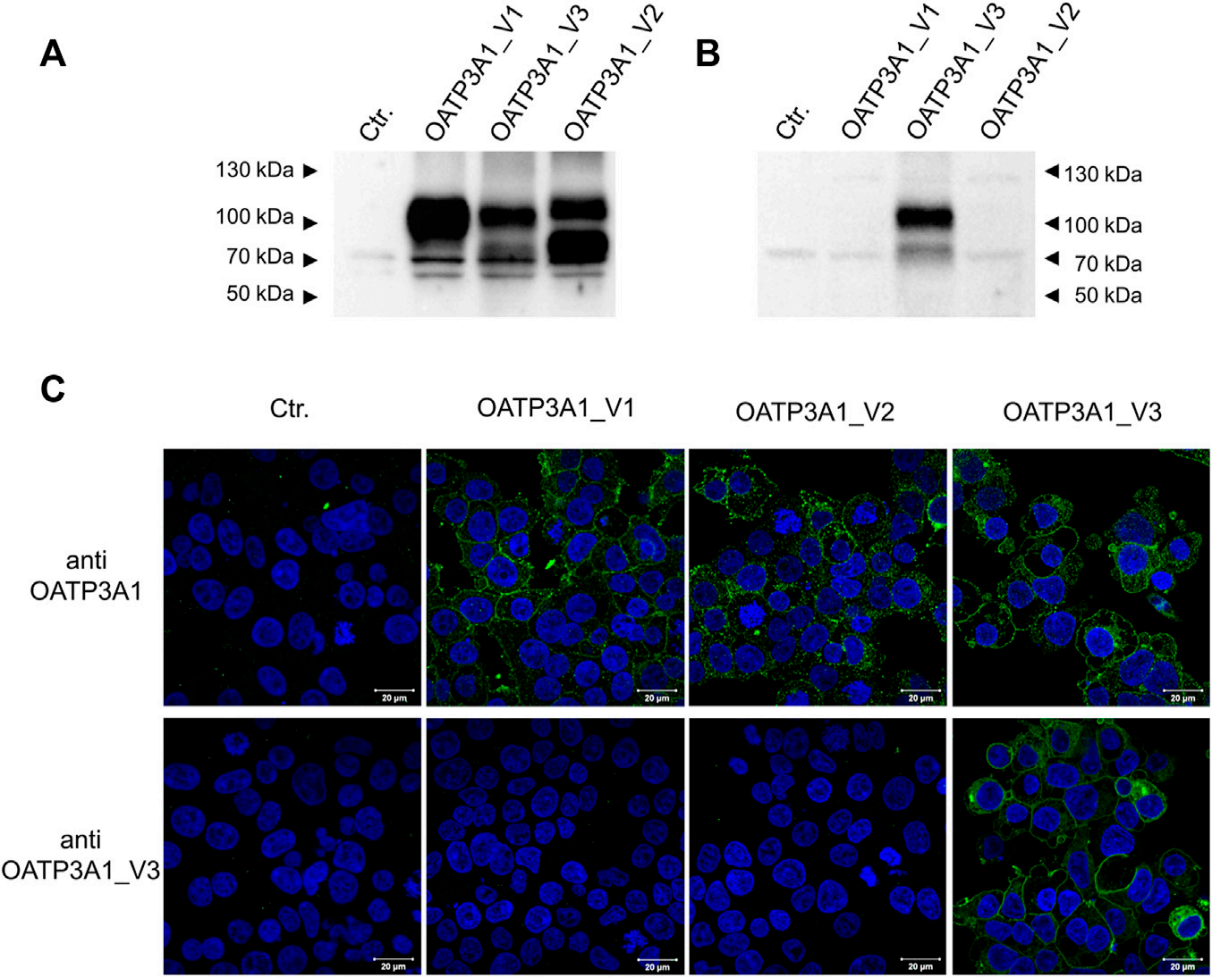
Specificity of the anti-OTP3A1_V3 antibody. **(A,B)**. Western blot analysis of HEK-293 cells overexpressing OATP3A1 variants. Western blot was performed on 5 μg of total cell lysates using the pan anti-OATP3A1 antibody (Sigma) **(A)** or the anti-OATP3A1_V3 antibody (described in Methods) **(B)**. Control (ctr.) represents mock-transfected HEK-293 cells. A representative blot of three independent experiments is shown. **(C)** Immunofluorescence staining of HEK-293 cells overexpressing OATP3A1 variants. Cells were fixed and labeled with the pan anti-OATP3A1 antibody (upper panel) or the anti-OATP3A1_V3 antibody (lower panel) (green). Nuclei were stained with DAPI (blue). Representative images are shown. Scale bar: 20 μm.

**FIGURE 4:**
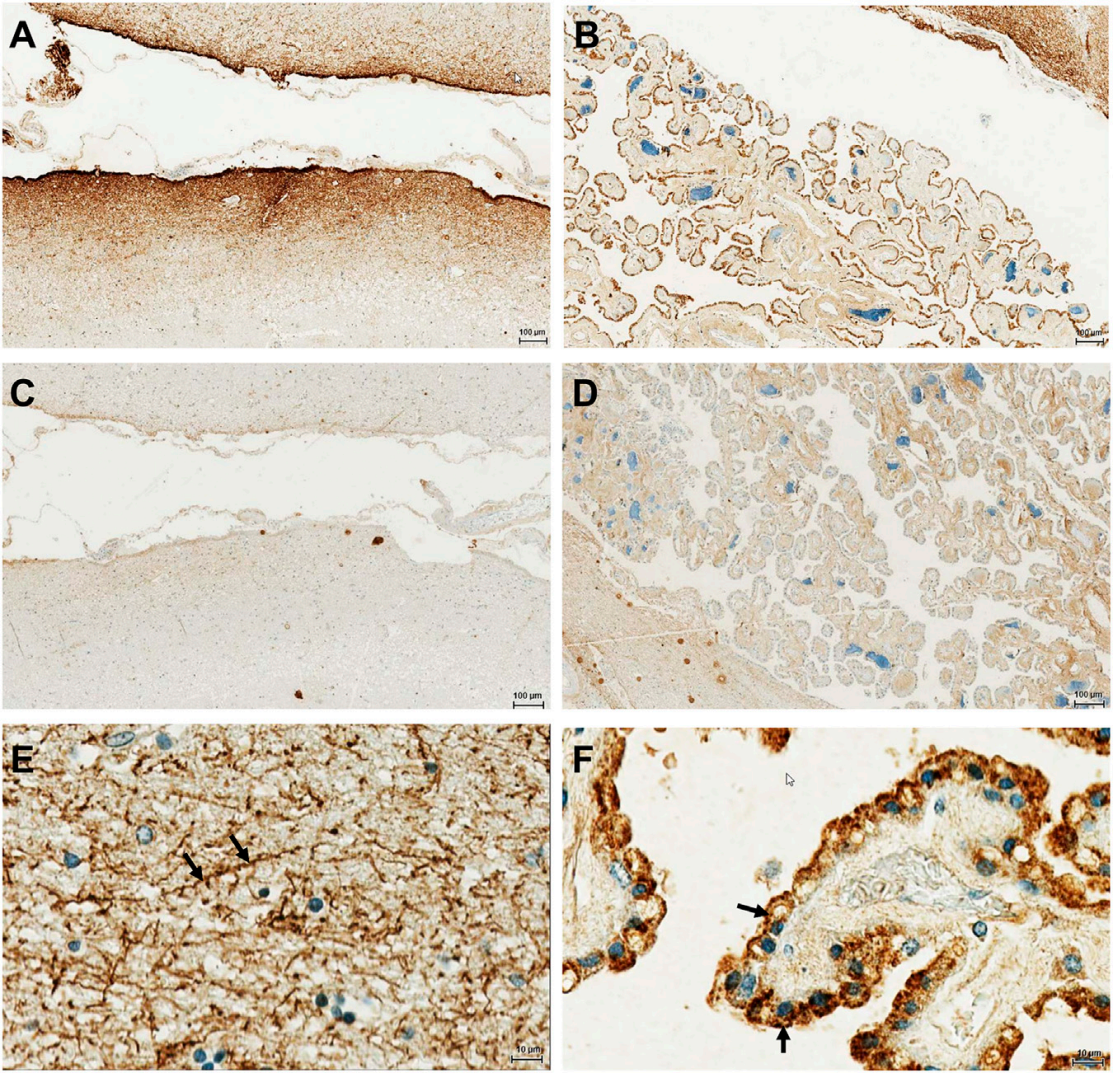
Immunohistochemical localization of OATP3A1_V3 in the human brain. The paraffin-embedded brain section was stained with the anti-OATP3A1_V3 antibody. **(A)**. Strong positive immunoreactivity was detected in the outer (subpial) cortex **(A)** and in the choroid plexus **(B)**. **(C,D)** Absence of staining after preincubation of the antibody with the peptide used for immunization (negative control). **(E)** Positive staining was observed in the brain cortex associated with neuronal axons (arrow). **(F)** Choroid plexus epithelium stained with the anti-OATP3A1_v3 antibody showing positive immunoreactivity at the apical (ventricular lumen facing) membrane (arrow). Nuclei were counterstained with hematoxylin.

**FIGURE 5:**
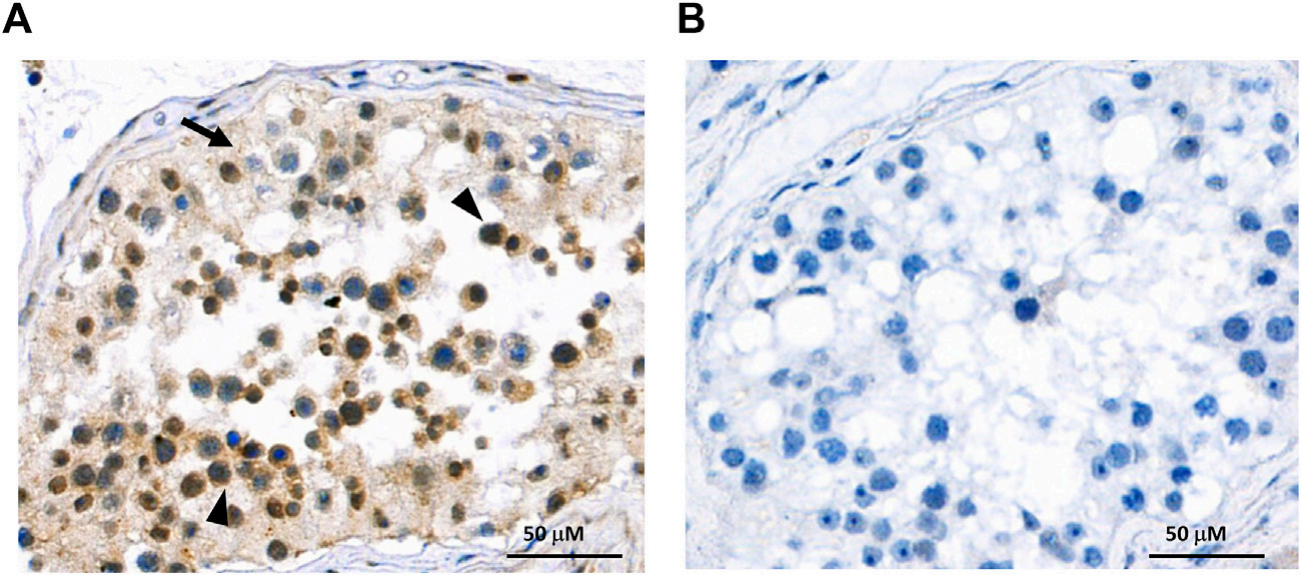
Immunohistochemical localization of OATP3A1_V3 in the human testis. **(A)** Paraffin-embedded testis section was stained with the anti-OATP3A1_V3 antibody and visualized by polymer-based detection. Positive staining was observed in germ cells (arrowhead) with limited labeling detected in Sertoli cells (arrow). Nuclei were counterstained with hematoxylin. **(B)** Negative control staining was performed in the absence of the primary antibody.

In order to investigate the protein expression of the new isoform in human tissues, a polyclonal antibody was generated against the peptide corresponding to the C-terminal 14 amino acids of the OATP3A1_V3 isoform (described in detail in *Materials and methods*). The specificity of the antibody was investigated by Western blot and immunofluorescence staining for which HEK-293 cells overexpressing one of the three OATP3A1 isoforms were generated (see details in the *Materials and methods* section and Figure 3). These experiments showed that the new polyclonal antibody specifically detected OATP3A1_V3 and did not cross-react with OATP3A1_V1 or OATP3A1_V2 in the overexpressing cell lines (although on the Western blot (Figure 3B), two weak, non-specific bands around 130 and 70 kDa could be detected). Then, immunolocalization studies with the novel antibody were performed in human brain and testis samples. As shown in Figures 4A,B strong signal was detected in the outer (subpial) cortex and choroid plexus. In neurons, OATP3A1_V3 localized to the axons (Figure 4E), and no immunolabeling of neuronal or glial cell bodies was observed. OATP3A1_V3 expression in the choroid plexus was restricted to the epithelial cells (Figure 4F) with strong labeling of the apical membrane. In testis, positive staining was observed in germ cells with weaker labeling detected in Sertoli cells (Figure 5).

### Localization of OATP3A1_V3 in MDCKII cells

**FIGURE 6:**
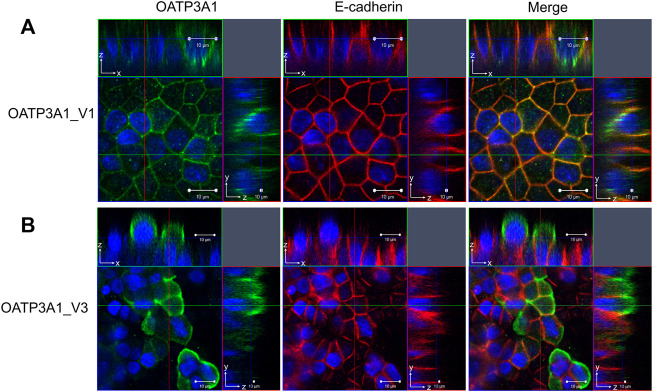
Subcellular localization of OATP3A1_V1 and OATP3A1_V3 in polarized MDCKII cells. MDCKII cells overexpressing OATP3A1 variants were grown on a permeable membrane support to form polarized cultures. Double immunostaining was performed with an antibody recognizing the lateral membrane marker E-cadherin (red) and the **(A)** pan anti-OATP3A1 antibody (OATP3A1_V1) or **(B)** anti-OATP3A1_V3 antibody (green). Right panels depict the overlay. OATP3A1_V1 is localized at the basolateral membrane and shows colocalization (yellow) with E-cadherin. Nuclei were stained with DAPI (blue). Optical XY sectioning with the corresponding XZ- or YZ-section was performed by confocal laser scanning microscopy. Representative images are shown.

Previously, Huber and colleagues demonstrated a different localization of OATP3A1_V1 and OATP3A1_V2 (Huber et al., 2007). In order to evaluate the subcellular expression of OATP3A1_V3 in polarized cells, MDCKII cells overexpressing OATP3A1_V1 or OATP3A1_V3 were generated. After polarization, the localization of OATP3A1 was investigated by immunofluorescence staining and confocal microscopy. Figure 6 shows that in harmony with previous results, OATP3A1_V1 localized to the lateral plasma membrane and co-localized with E-cadherin. In contrast, OATP3A1_V3 was detected in the apical/subapical plasma membrane of polarized MDCKII cells.

### Functional analysis of OATP3A1_V3

**FIGURE 7:**
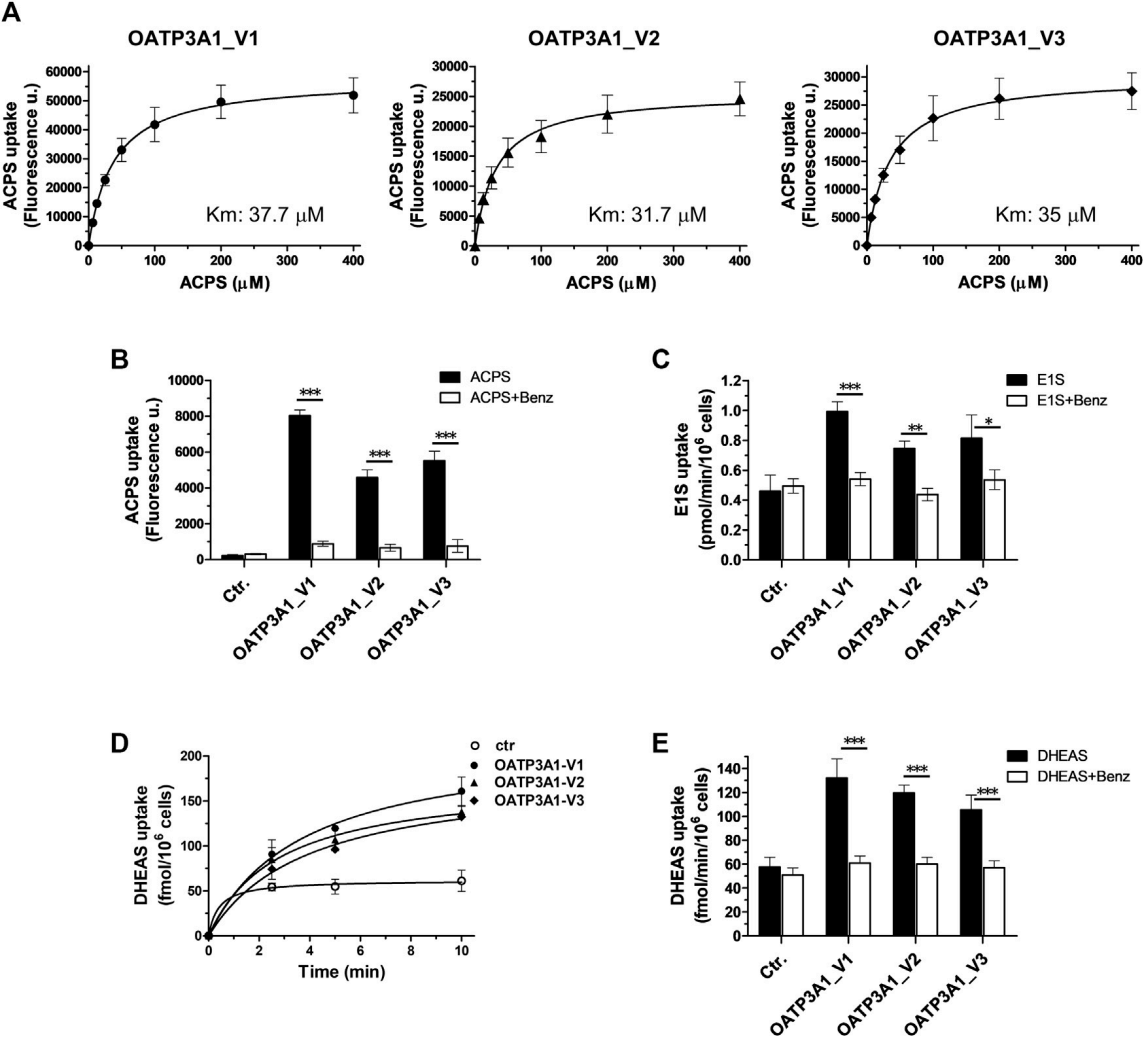
Transport activity and inhibitor sensitivity of transport in HEK-293 cells overexpressing OATP3A1 variants. **(A)**. Concentration dependence of ACPS uptake by OATP3A1 variants. HEK-OATP3A1 cells were incubated with 5–400 µM ACPS for 10 min. Transport data were obtained by subtracting the fluorescence in mock cells from that measured in HEK-OATP3A1 cells. **(B)**. Inhibition of ACPS uptake (0.5 μM, 10 min) by benzbromarone (Benz, 20 µM). **(C)** ^3^H -E1S uptake (5 µM, 10 min) by OATP3A1 variants in the presence or absence of benzbromarone (20 µM). **(D)**. Time course of ^3^H -DHEAS uptake by OATP3A1 variants. Cells were incubated with 0.5 µM ^3^H -DHEAS for the indicated time. **(E)**. Inhibition of ^3^H -DHEAS uptake (0.5 µM, 5 min) by benzbromarone (20 µM).

Finally, functionality of the novel isoform was examined by measuring the uptake of the established OATP3A1 substrates, E1S and the fluorescent dye ACPS ((E)-1-(3-azidopropyl)-4-(2-(7-(diethylamino)-2-oxo-2H-chromen-3-yl)vinyl)pyridine-1-ium-3-sulfonate)) (Bakos et al., 2020). We found a saturable, inhibitor-sensitive uptake of ACPS in HEK-293 cells overexpressing OATP3A1_V1, OATP3A1_V2, and OATP3A1_V3 (Figures 7A,B). The affinity toward the fluorescent dye of the three isoforms was similar (Km V1: 37.7 μM, Km V2: 31.7 μM, and Km V3: 35 μM). ACPS transport could be inhibited by benzbromarone, a known inhibitor (Bakos et al., 2020). In radioligand-based assays, we also confirmed the functionality of the novel isoform using E1S (Figure 7C), a transported substrate. Finally, overexpression of OATP3A1_V1, OATP3A1_V2, or OATP3A1_V3 resulted in a saturable, inhibitor-sensitive uptake of DHEAS into HEK-293 cells (Figures 7D,E), identifying DHEAS as a novel substrate of OATP3A1.

## Discussion

OATP3A1 is a ubiquitously expressed plasma membrane transporter (Tamai et al., 2000), mediating the cellular uptake of prostaglandins, vasopressin, E1S, and several drugs (Bakos et al., 2020, Huber et al., 2007, Atilano-Roque and Joy, 2017). OATP3A1 is evolutionally conserved, suggesting essential physiological function(s), one of which is the transport of neuropeptides in the central nervous system (Huber et al., 2007). In addition, although OATP3A1 is not generally considered as a major hepatic bile acid transporter (Dawson et al., 2009), OATP3A1 can potentially mediate bile acid efflux from hepatocytes in cholestatic liver (Pan et al., 2018).

In addition to the first cloned and characterized OATP3A1, corresponding to OATP3A1_V1 (isoform 1) (Tamai et al., 2000), the NCBI database contains additional entries of which only isoform 2 (OATP1B3_V2) arising due to alternative splicing has been previously verified and characterized (Huber et al., 2007). Huber and colleagues demonstrated wide tissue distribution of both OATP3A1_V1 and OATP3A1_V2, and the two isoforms were also similar in their substrate recognition. However, the expression patterns of the two isoforms were different. Compared to OATP3A1_V2, OATP3A1_V1 showed generally 10-times higher expression in all tissues. In addition, trafficking of the two isoforms was different in choroid plexus cells, with apical localization of OATP3A1_V2 and basolateral localization of OATP3A1_V1.

Here, we demonstrate the existence and function of a third OATP3A1 isoform, X1 (XP_005254946.1), for which we recommend OATP3A1_V3 as the designation. The entire protein coding sequence of *SLCO3A1*-V3 mRNA could be amplified from the brain RNA library, and based on qPCR, is ubiquitously expressed (Figure 2). However, similar to OATP3A1_V2, mRNA expression levels of OATP3A1_V3 were an order of magnitude lower than those of OATP3A1_V1. Therefore, although mRNA levels do not necessarily correlate with protein expression, in those cells where both OATP3A1_V1 and OATP3A1_V3 are present, OATP3A1_V3 may have lower relevance. The exception can be the testis, with similar OATP3A1_V1 and OATP3A1_V3 expressions. In addition, according to our results (Figure 5A) and those by Huber et al. (2007), in Sertoli cells, OATP3A1_V3 (and OATP3A1_V2) may be dominant. The unique C-terminus of OATP3A1_V3 allowed the generation of an isoform-specific antibody (Figure 3), with which we could show the protein expression of OATP3A1_V3 in the brain (neurons and choroid plexus) and testis (Figures 4, 5). Based on immunolocalization studies (Figures 4, 5), we found that the expression pattern of OATP3A1_V3 differs from that of OATP3A1_V1 (Huber et al., 2007). In choroid plexus epithelial cells, OATP3A1_V3 localized to the apical membrane, which is similar to that earlier observed for OATP3A1_V2 (Huber et al., 2007). However, weak intracellular staining of OATP3A1_V3 could also be observed in choroid plexus epithelial cells and also in HEK-293 or MDCKII cells (Figure 3C and Figure 6B). In testis, we found a wider expression of OATP3A1_V3, different from the expression of OATP3A1_V1 restricted to germ line cells (Huber et al., 2007). Distinct localization of OATP3A1_V1 and OATP3A1_V3 was also confirmed in polarized MDCKII cells overexpressing these isoforms (Figure 6).

Most OATPs are plasma membrane proteins; however, e.g., OATP2A1 is localized in lysosomes (Shimada et al., 2015). In polarized cells, OATPs can be found either in the apical or in the basolateral membrane. OATP1A2 is apically localized in brain endothelial cells (Gao et al., 2000), while OATP1B1 and OATP1B3 are localized in the basolateral (sinusoidal) membranes of hepatocytes (Konig et al., 2000, Kullak-Ublick et al., 2001). Localization of OATP2B1 in hepatocytes was demonstrated in the basolateral membrane, although data regarding trafficking in enterocytes are controversial (McFeely et al., 2019). Cellular routing of plasma membrane transporters in polarized epithelial cells is governed by diverse mechanisms and may vary between cell types (Stoops and Caplan, 2014). Signals and motifs determining the cellular routing of OATPs have not yet been fully understood. N-glycosylation, phosphorylation, various transmembrane regions, or the N-terminus have already been shown to regulate the plasma membrane trafficking of OATPs (Lee et al., 2020). However, to date, only the PDZ-binding motif has been shown to regulate the apical routing of OATP1A2 and OATP2B1 (Zheng et al., 2014, Ferreira et al., 2018). Interestingly, interaction of the isolated C-terminal peptide OATP3A1 with PDZ proteins NHERF1, NHERF2, and IKEPP (NHERF4) was demonstrated earlier using a yeast two-hybrid system (Kato et al., 2004). Unfortunately, the authors did not clarify the exact sequence of the C-terminal peptide they used. Notably, of the three OATP3A1 isoforms, only OATP3A1_V1 contains a C-terminal PDZ-binding motif. NHERF is expressed in epithelial cells and recruits its binding partners to or close to the apical membrane (Voltz et al., 2001); therefore, an apical routing of OATP3A1_V1 would be expected. In contrast, OATP3A1_V2 and OATP3A1_V3, which lack a C-terminal PDZ-binding motif, are localized apically, while OATP3A1_V1 is found basolaterally. These findings suggest that trafficking of OATP3A1 is not governed (solely) by PDZ interaction, although further studies are required to clarify this issue.

Several isoforms of human OATP protein family members formed either by alternative transcription initiation or alternative splicing have already been characterized. Alternative transcription initiation results in five OATP2B1 transcripts (a-e) and two different polypeptides. OATP2B1 isoforms a, c, d, and e encode a 709-amino acid long polypeptide, while isoform b results in an N-terminally truncated (687 aa), but still functional OATP2B1. Albeit with slightly different expression patterns, the two OATP2B1 protein versions were not different in their function or plasma membrane localization (Knauer et al., 2013). In the case of OATP1B3, a liver-specific multispecific organic anion transporter, alternative transcription initiation results in a shorter mRNA and an N-terminally truncated isoform, ct-OATP1B3-V1 (Imai et al., 2013, Nagai et al., 2012). Ct-OATP1B3-V1 is characteristic of cancerous cells and is not expressed in healthy tissues (Furihata et al., 2015, Sun et al., 2014). In contrast, expression of the liver-type OATP1B3 is almost exclusively restricted to healthy hepatocytes (Furihata et al., 2015, Konig et al., 2000). Some researchers suggested that the shorter ct-OATP1B3-V1 isoform is trapped inside the cells (Sun et al., 2014, Thakkar et al., 2013), while others found it to be a fully functional plasma membrane transporter (Imai et al., 2013).

Here, we demonstrated the functionality of OATP3A1_V3 using the artificial substrate fluorescent dye ACPS and the endogenous substrate steroid hormone precursors E1S and DHEAS (Figure 7). DHEAS is a neuroactive steroid and a novel substrate for all three OATP3A1 isoforms. Since OATPs work as exchangers and the efflux of substrates by OATP3A1 has been demonstrated, the expression pattern and different localization of OATP3A1_V1 and OATP3A1_V3 may suggest a concerted action of these isoforms in the transepithelial movement of neurosteroids. However, further studies are required, including a detailed characterization of the substrate specificity of isoform OATP3A1_V3.

Increased expression of OATP3A1 was shown in breast, pancreas and liver tumors (Banerjee et al., 2015, Hays et al., 2013, Kindla et al., 2011, Wlcek et al., 2011), although the isoforms in these studies have not been investigated. Nevertheless, considering steroid uptake mediated by all three isoforms, one may speculate that their presence may increase tumor progression.

In conclusion, in this investigation, we verified the expression and functionality of a novel OATP3A1 isoform expressed in various organs, including the choroid plexus. OATP3A1_V3 together with several other members of the OATP family, OATP1A2, OATP1Bs, and OATP2B1 and the other two OATP3A1 isoforms, may influence steroid hormone homeostasis.

## Data Availability

Data will be available upon request made to the corresponding author.

## Abbreviations

ACPS, (E)-1-(3-azidopropyl)-4-(2-(7-(diethylamino)- 2-oxo-2H-chromen-3-yl)vinyl)pyridine-1-ium-3-sulfonate); DHEAS, dehydroepiandrosterone sulfate; E1S, estrone-3-sulfate; OATP, organic anion transporting polypeptide.
